# Supplementary material for: On the linear in probability model for binary data
Source: R Soc Open Sci. 2019 May 1;6(5):190067. doi: 10.1098/rsos.190067 (PMC6549984; doi:10.1098/rsos.190067)
Supplement: Data and variables [file rsos190067supp1.pdf]

Appendix

Data and variables

Our data are drawn from the National Longitudinal Study of Youth 1979. The full NLSY dataset can be downloaded for free from https://www.nlsinfo.org/ (site registration required). We selected the following variables:

Ability **afqt3 (R0618301)**

Individual's AFQT score, measured in the 1981 survey. Scores were renormed and revised in 2006 (see https://www.nlsinfo.org/sites/nlsinfo.org/files/attachments/140116/AFQT%20Analysis%20Results%20Final%20Revised.pdf for details of AFQT measures).

College enrollment **hgrev98 (R64796)** **hgcrev89 (R30748.01)**

We measure the highest grade of education completed by the individual, captured in the 1998 survey. We recode the variable to distinguish between those who did not enroll in college (i.e. completed 12th grade or lower), and those who completed at least one year of college. The data on education were collected just under twenty years after the first wave of the survey, and missing data are therefore introduced via dropout. Where data are missing, we use information on highest grade provided in the 1989 survey.

Family income **tnfi_ trunc (R02179.00)**

Net income of individual's family, measured in the 1979 survey.

NLSY sampled individuals aged 14-22. As a result, the sample contains individuals who are still dependent upon parents and other family members, individuals who are independent of parents and living alone, and individuals who are independent of parents and established in a household with other adults (and perhaps children). The family income variables were not asked of individuals living alone. Missing data on family income, then, comes from three main sources:

1. Individuals who provided income data, but the data pertains to their own household.
2. Individuals who were not included in these data because they live alone.
3. Missing data arising from refusal to provide income data (e.g. "don't know", or "refused").

Our interest is in the effect of family income in childhood on child outcomes, so we restrict the income measurement to individuals aged 18 and under (using the variable **fam-1b (R0000600)**). Income data are treated as missing for individuals aged 19 and over.

Living with parents **fam-20 (R0007100)** **fam-27 (R0008500)**

We construct a variable that captures whether or not the respondent was living with at least one parent at the time of the survey (using variables **fam-20 (R0007100)** and **fam-27 (R0008500)**). These variables were measured in the 1979 survey.

Gender **sample_sex (R0214800)**

Individual's self-classified gender (male or female), measured in 1979 survey.

No missing cases. No data cleaning/manipulation.

Race **sample_race (R02147)**

Individual's race, as classified by interviewer, measured in 1978 screener.

No missing cases. All individuals classified as "Hispanic" excluded from the analysis.
